# Selection of Reference Genes for Quantitative Real-Time PCR in Bamboo (*Phyllostachys edulis*)

**DOI:** 10.1371/journal.pone.0056573

**Published:** 2013-02-20

**Authors:** Chunjie Fan, Jinmin Ma, Qirong Guo, Xiaotie Li, Hui Wang, Mengzhu Lu

**Affiliations:** 1 State Key Laboratory of Tree Genetics and Breeding, Research Institute of Forestry, Chinese Academy of Forestry, Beijing, People’s Republic of China; 2 Research Institute of Tropical Forestry, chinese Academy of Forestry, Guangzhou, People’s Republic of China; 3 Beijing Genomics Institute-Shenzhen, Shenzhen, People’s Republic of China; 4 International Centre for Bamboo and Rattan, Beijing, People’s Republic of China; 5 Guilin Research Institute of Forestry, Guilin, People’s Republic of China; 6 Centre for Ecology and Hydrology, Natural Environment Research Council, Wallingford, United Kingdom; Instituto de Biología Molecular y Celular de Plantas, Spain

## Abstract

**Background:**

The Moso bamboo (*Phyllostachys edulis*) is one of the most important forestry resources and plays essential ecological roles in southern China. A draft nuclear genome sequence is expected to be publicly available in the near future; an explosion of gene expression data related to the unique traits of Moso bamboo will undoubtedly follow. Reverse transcription quantitative real-time PCR ((RT-)qPCR) is a widely used method for gene expression analysis. A necessary prerequisite of exact and reliable data is the accurate choice of reference genes.

**Result:**

In this study, 14 candidate reference genes were chosen, and their expression levels were assessed by (RT-)qPCR in a set of six tissue samples (root, stem, mature stem, leaf, flower, and leaf sheath) and at two developmental stages (before and after flowering) in bamboo specimens obtained in three locations. The stability and suitability of the candidate reference genes were validated using the geNorm, NormFinder and BestKeeper programs. The results showed that *TIP41* and *NTB* were suitable reference genes across all the tissues and at the different developmental stages examined in this study. While the expression of the *NTB, TIP41* and *UBQ* were the mostly stable in different plant tissues samples, the expression of the *TIP41, NTB* and *CAC* were ranked the most stable in bamboo plants at various developmental stages. *AP2-like* gene was further assessed by using the reference genes *TIP41* and *NTB* in comparison to *ACT*. Significant difference of the expression profile of *AP2-like* demonstrated the importance of choosing adequate reference genes in bamboo.

**Conclusion:**

*TIP41* and *NTB* were found to be homogeneously expressed and were adequate for normalization purposes, showing equivalent transcript levels in different samples. They are therefore the recommended reference genes for measuring gene expression in *P. edulis*.

## Introduction

Bamboo, a perennial monocot classified in the subfamily Bambusoideae within the family Poaceae that includes rice, maize, wheat and other cereals [1], is one of the most important forest resources. The Moso bamboo (*P. edulis*) has a rather striking speed of growth with a final height of more than ten meters within a short period of two to four months [2]. Apart from these striking features, it is surprising that the vegetative phase of Moso bamboo can last up to 100 years or longer before flowering and that the plants die after flowering [3]. With these unique features, the Moso bamboo is the most widely distributed bamboo species with the largest planting area and has the highest economic value in China [4]. Biochemical, physiological and cytogenetic studies of this plant have been carried out for several decades [5], [6], whereas the analysis of genes, and their transcription and expression in bamboo has been rare and slow, since most of the genes are unknown. To accelerate genetic studies in bamboo, the entire bamboo genome has recently been sequenced (Lubin Li, personal communication) and the partial contigs have been already deposited in the NCBI database (http://www.ncbi.nlm.nih.gov). This development is expected to facilitate significant progress in research into the molecular biology of this species.

Gene expression analysis is increasingly important in many fields of plant biological research including in development and responses to abiotic stress and infection by pathogens. (RT-)qPCR is currently used extensively to study gene expression changes due to its high sensitivity and specificity, and reliable quantification with this method depends on the use of stably expressed endogenous genes as reference genes [7], [8]. Thus, it is necessary to first study the stability of expression of several endogenous genes in order to select a suitable internal reference that is expressed constitutively across treatments, tissues and developmental stages. In the last several years, there has been a rapid increase in reports focused on stability analysis of reference genes in various conditions, development stage and organisms in plants [9], [10], [11], [12]. However, a systematic study validating reference genes has not been performed in bamboo (*P. edulis*). Until now, *ACTIN (ACT)* has been used as the internal reference gene in gene expression analysis by (RT-)qPCR and semi-quantitative PCR in bamboo. Although *ACT*s are stably expressed in different tissues of several plant species [12], [13], this may not be true in all plant species. For instance, *ACT* was not a suitable reference gene *in* one of grass species, *Brachypodium distachyon,* at different developmental stages and under various experiment conditions [14]. Even in rice, the expression of *ACT* was not detected in all 3 biological replicates of the semi apical meristem [11]. Therefore, it is necessary to validate the expression stability of the internal control gene in the target species of interest.

In this report, the stability of fourteen candidate reference genes for use in bamboo gene expression studies was examined. Genes including *ACT,* glyceraldehyde-3-phosphate (*GAPDH*), elongation factor 1-alpha (*EF1α*), alpha tubulin (*TUBα*), tubulin beta-1 chain (*TUBβ*), ribosomal protein 18 s (*18S*) *and* ubiquitin (*UBQ*), whose stability has been analyzed in other plants, are commonly employed as references in gene expression studies. In addition to the genes mentioned above, ubiquitin-conjugating enzyme (*UBC*), clathirin adaptor complexes medium subunit (*CAC*), peptidyl-prolyl cis-tans isomerase/cyclophilin (*CYP*), endo-1,3-beta-glucanase (*GLU*), malate dehydrogenase in cytosol (*MDH*), tonoplast intrinsic protein (*TIP41*), ubiquitin-conjugating enzyme E2 36-like (*UBC*) and nucleotide tract-binding protein (*NTB*), whose expression was reported to be stable [8], [11], were also included as candidate reference genes in bamboo.

## Results

### Expression Profiles of Reference Genes

**Figure 1:**
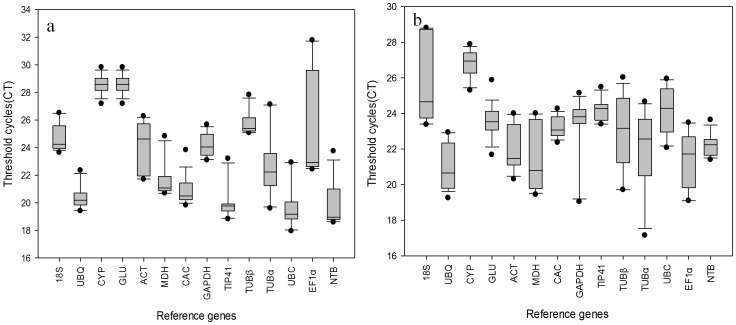
Absolute Ct values in qPCR of samples. (a) Different tissues, (b)Different developmental stages grown in three geographical locations. Each box indicates 25/75 percentiles. Whisker caps represent 10/90 percentiles. The median is depicted by the line and the dots represent outliers of 10th/90th percentiles.

A total of 14 genes were selected as candidates for internal controls, for normalization of gene expression measures. They were *18* *s*, *UBQ*, *CYP*, *GLU*, *ACT*, *MDH*, *CAC*, *GAPDH*, *TIP41*, *TUBα*, *TUBβ*, *UBC*, *EF1α* and *NTB.* A (RT-)qPCR assay based on SYBR Green dye detection was carried out to examine the stability of the expression of the 14 candidate genes (Table 1). For each gene, the full sample set was included in each technical replicate to exclude any artifacts due to variations between runs. Each sample was measured in triplicate within each run, and three independent technical replicates were performed for each experiment. The expression levels of the candidate reference genes were presented as the quantification cycle (Cq) value, representing the cycle at which a significant increase of the PCR product occurs (Figure 1). The expression levels of these 14 reference genes varied widely, with Cq values ranging from 18 to 30 cycles, with most of the Cq values falling between 20 and 25 cycles. *TUBα* was the most highly expressed of the set, with a mean Cq value of 19.7. *CYP* showed the lowest level of expression in all samples, with Cq values as high as 28.6 cycles among different tissues samples and 27.1 cycles in samples from different developmental stages. The calculated coefficient of variance (CV) of the Cq values indicated the expression stability of a particular gene. The narrower the range of the CV value, the more stably the given gene was expressed in different samples. Among the 14 candidate reference genes in this study, *TUBβ*, whose CV value was more than 6 cycles, showed a much greater variation in expression levels than the other genes in different tissue samples. The proportion of *CAC, NTB* and *TIP41* transcripts, with CV values of 1.9, 2.01 and 1.7, respectively, remained relatively constant in samples from different developmental stages, but the opposite was true for *TUBβ* and *EF1α*, both in different tissues and at different development stages. It could be seen that none of the transcript levels of the reference genes was truly constant. Therefore, it is essential to select a set of reliable reference genes to normalize gene expression to obtain accurate gene expression data.

**Table 1:** Genes and primer sets used for real time RT-PCR.

Gene abbreviation	Genbank accession	Rice ortholog locus	Description	Primer pair (forward/reverse)	Product size (bp)	Efficiency
GAPDH	gi|242389794	Os08g03290	Glyceraldehyde-3-phosphate dehydrogenase	CTCTTCGGCCAGAAGCCAGTCAC TTGGCACCACCCTTCAAGTGAGC	149	99.64%
EF1α	gi|242386476	Os03g08050	Elongation factor 1-alpha	AAGGCTGAGCGTGAAAGAGGTAT AATGACCGTGCAGTAGTATTTGG	81	99.11%
MDH	gi|242384436	Os10g0478200	Malate dehydrogenase, cytosolic	GCACATGCTTGATATTCCACCAG CAACCATAACCGCAACATTCACA	149	100.59%
UBC	gi|242387089	Os01g0673600	Ubiquitin-conjugating enzyme E2 36-like	TCTTCAGATACGCACAGTTCTTT CAGCTTCAGCTTCATTGGCTTTC	116	95.37%
UBQ	gi|242386091	Os02g16040.1	Ubiquitin	GGGTCGTCCAGTGTCCTCTATTA TCAACCAAACCACTGTACCTCAG	133	96.23%
18S	gi|242375117	Os01g0610500	18s ribosomal RNA	CGGCTACGTGACATTTGTTCCTC AATCGACAGAAGCGATGCTGGAA	136	91.05%
ACT	gi|301071262	Os05g0438800	Actin 1	ATACGCTTCCTCACGCTATTCTT CCGAGCTTCTCCTTTATGTCCCT	145	99.25%
TUBα	gi|242385991	Os03g0726100	Alpha tubulin	TGACATTGAGCGCCCAACTTACA ATCCACATTCAGAGCACCATCGA	103	99.86%
TUBβ	gi|242382054	Os05g0413200	Tubulin beta-1 chain	TCTTCCCTTCGCCAAAGGTCTCA CTCATCAGCATTCTCCACCAACT	89	97.21%
TIP41	gi|242384689	Os03g0760600	TIP41-like family protein	AAAATCATTGTAGGCCATTGTCG ACTAAATTAAGCCAGCGGGAGTG	102	100.55%
CYP	gi|242381616	Os09g0571400	Peptidyl-prolyl cis-transisomerase/cyclophilin	GGTCGGAGAAATCCGTCAAGAGG CTTGCCGATCAGGATGTCGAAGA	116	99.62%
GLU	gi|242386432	Os01g0946600	Endo-1,3-beta-glucanase mRNA	GGTATAACCAGGGCCTGATTGAC TTAGGGTAGAAGAGCCCGAAGTG	145	100.05%
CAC	gi|242375393	Os12g0207300	Clathrin adaptor complexes medium subunit	AGTGAAACCGTTCCTTCCTCTGC AGAACAATCTGCCAGTAACCTCA	157	98.35%
NTB	gi|242381788	Os05g0437300	Nucleotide tract-binding protein	TCTTGTTTGACACCGAAGAGGAG AATAGCTGTCCCTGGAGGAGTTT	133	96.05%
AP2-like	gi|242374576	Os05g0121600	APETALA-2 transcription factor	CAAGCAACCAAGCAAACAGA CCCGATCAACACCTCTCACT	137	–

### GeNorm Analysis

**Figure 2:**
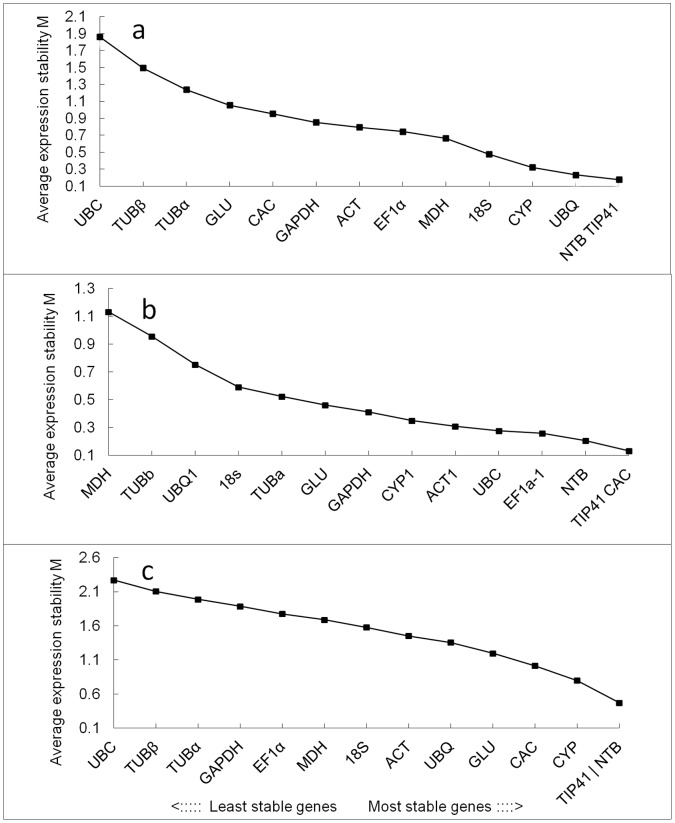
Average expression stability and ranking of 14 candidate reference genes as calculated by geNorm. (a) Different tissues, (b) Different development stages grown in three geographical locations, (c) All samples. Lower average expression stability (M value) indicates more stable expression.

The geNorm software is employed as a means of determining the expression stability of the selected reference genes (http://medgen.ugent.be/~jvdesomp/genorm/) [15]. The program is a Visual Basic application (VBA) tool for Microsoft Excel and relies on the principle that the expression ratio of two perfect reference genes should be constant under different conditions or in various plant tissues. It calculates a measure of the expression stability (M) based on the average pairwise variation between all reference genes tested (Figure 2). The gene with the lowest M value is considered to have the most stable expression, while that with the highest M value has the least stable expression. In this study, the *NTB* and *TIP41* genes were most stably expressed in different plant tissue samples, with an M value of 0.179, while *UBC* was the least stably expressed, with an M value of 1.9 (Figure 2a). For the samples in vegetative and reproductive growth stages derived from different geographical locations, *TIP41* and *CAC* genes ranked high, indicating that these genes were stably expressed and probably played a housekeeping role (Figure 2b). When all the samples were considered together, as shown in Figure 2c, the results remained very similar in different tissues; the average expression stability values (M) of *TIP41* and *NTB* were the lowest, and that of *UBC* was the highest, indicating that *TIP41* and *NTB* had the most stable expression and that *UBC* was expressed most variably.

### NormFinder Analysis

The Normfinder software (http://www.mdl.dk/publicationsnormfinder.html), another VBA applet, which is based on a variance estimation approach, ranks the genes according to their stability under a given set of experimental conditions, and more stable gene expression is indicated by lower average expression stability values [16]. We used this for further confirmation of the results obtained with the geNorm program. The results showed that the ranking of the stability of reference genes with NormFinder was slightly different from the one determined by geNorm (Table 2). The *UBQ* gene ranked highest for different plant tissue samples, followed by *NTB, TIP41* and *MDH*, while the *TIP41* and *CAC* genes appeared to be the highest reliable controls for plant samples at different developmental stages. When considering all the experimental samples, *CAC* genes was identified as the most reliable gene, *UBQ, GLU, ACT* and *TIP41* followed and were almost of the same stability, with M values of 1.129, 1.132, 1.146 and 1.159, respectively.

**Table 2:** Expression stability values for bamboo candidate reference genes calculated using Normfinder.

Rank	Tissues	Developmentalstages	Total
1	UBQ (0.504)	TIP41 (0.701)	CAC (0.935)
2	NTB (0.690)	CAC (0.722)	UBQ (1.129)
3	TIP41 (0.719)	GLU (0.846)	GLU (1.132)
4	MDH (0.854)	UBC (0.858)	ACT (1.146)
5	ACT (0.857)	NTB (0.915)	TIP41 (1.159)
6	CAC (0.899)	CYP (1.066)	NTB (1.242)
7	GAPDH (0.916)	EF1α (1.381)	CYP (1.341)
8	CYP (0.992)	ACT (1.442)	MDH (1.590)
9	18S (1.019)	UBQ (1.529)	18S (1.673)
10	GLU (1.283)	GAPDH (1.751)	EF1α (1.739)
11	TUBα (1.452)	MDH (2.099)	TUBα (1.943)
12	EF1α (1.583)	TUBα (2.144)	GAPDH (2.118)
13	TUBβ (2.356)	18S (2.146)	TUBβ (2.243)
14	UBC (3.968)	TUBβ (2.250)	UBC (2.879)

Note: Expression stability and ranking of 14 candidate reference genes calculated with NormFinder in all samples, plant tissues and developmental stages. Lower average expression stability (M value) indicates more stable expression.

### BestKeeper Analysis

The BestKeeper program is another Excel-based software tool to analyze the stability of a candidate reference gene, based on the coefficient of variance (CV) and the standard deviation (SD) of the Cq values, by using the average Cq value of each duplicate reaction [17]. Reference genes are identified as the most stable genes when they exhibit the lowest coefficient of variance and standard deviation (CV ± SD). In this study, the BestKeeper analysis revealed that *CAC* and *TIP41* had CV values of 2.68±0.64 and 3.06±0.77, respectively (Table 3), and showed remarkably stable expression in all the samples. However, *GAPDH* and *TUBα* had CV values of 9.89±2.14 and 9.16±1.88, respectively, and showed the least stable expression. Among the different tissues of bamboo, the most stable reference genes were *CYP* and *TIP41*, with the lowest CV values, while *UBC* had the highest CV value of all the selected genes. Bestkeeper analyses indicated that *TIP41* and *NTB* were the most stably expressed genes, and *TUBα* and *TUBβ* were the least stably expressed genes for plant samples derived from different geographical locations at different developmental stages. When all the experimental samples were evaluated, *TIP41, NTB* and *CAC* were identified as the appropriate reference genes by the three programs, although their rank orders were slightly altered between programs.

**Table 3:** Expression stability values for bamboo candidate reference genes calculated using BestKeeper.

Rank	Tissues	Developmentalstage	Total
1	CYP (1.84±0.53)	TIP41 (1.69±0.41)	CAC (2.68±0.64)
2	TIP41 (2.58±0.67)	NTB (2.16±0.48)	TIP41 (3.06±0.77)
3	NTB (2.77±0.66)	CAC (2.36±0.55)	CYP (3.35±0.93)
4	CAC (3.01±0.73)	CYP (2.79±0.76)	NTB (3.61±0.84)
5	18S (3.24±0.81)	GLU (2.86±0.68)	UBQ (4.74±0.99)
6	UBQ (3.42±0.7)	UBC (3.55±0.87)	GLU (4.84±1.16)
7	MDH (3.86±0.81)	EF1α(5.36±1.18)	ACT (5.66±1.25)
8	GAPDH (4.33±0.87)	UBQ (5.51±1.17)	18S (5.69±1.44)
9	ACT (4.77±1.04)	GAPDH (5.65±1.31)	MDH (6.22±1.33)
10	TUBα (5.89±1.16)	ACT (6.25±1.39)	EF1α (7.99±1.68)
11	GLU (6.17±1.5)	MDH (7.86±1.71)	TUBβ (8.03±1.83)
12	EF1α (7.10±1.42)	18S (7.90±2.03)	UBC (8.44±2.11)
13	TUBβ (7.82±1.77)	TUBβ (8.18±1.87)	TUBα (9.16±1.88)
14	UBC (13.61±3.47)	TUBα(8.85±1.89)	GAPDH (9.89±2.14)

Note: Reference genes are identified as the most stable genes, as assessed by the lowest values of the coefficient of variance (CV) and standard deviation (SD).

### Reference Gene Validation

**Figure 3:**
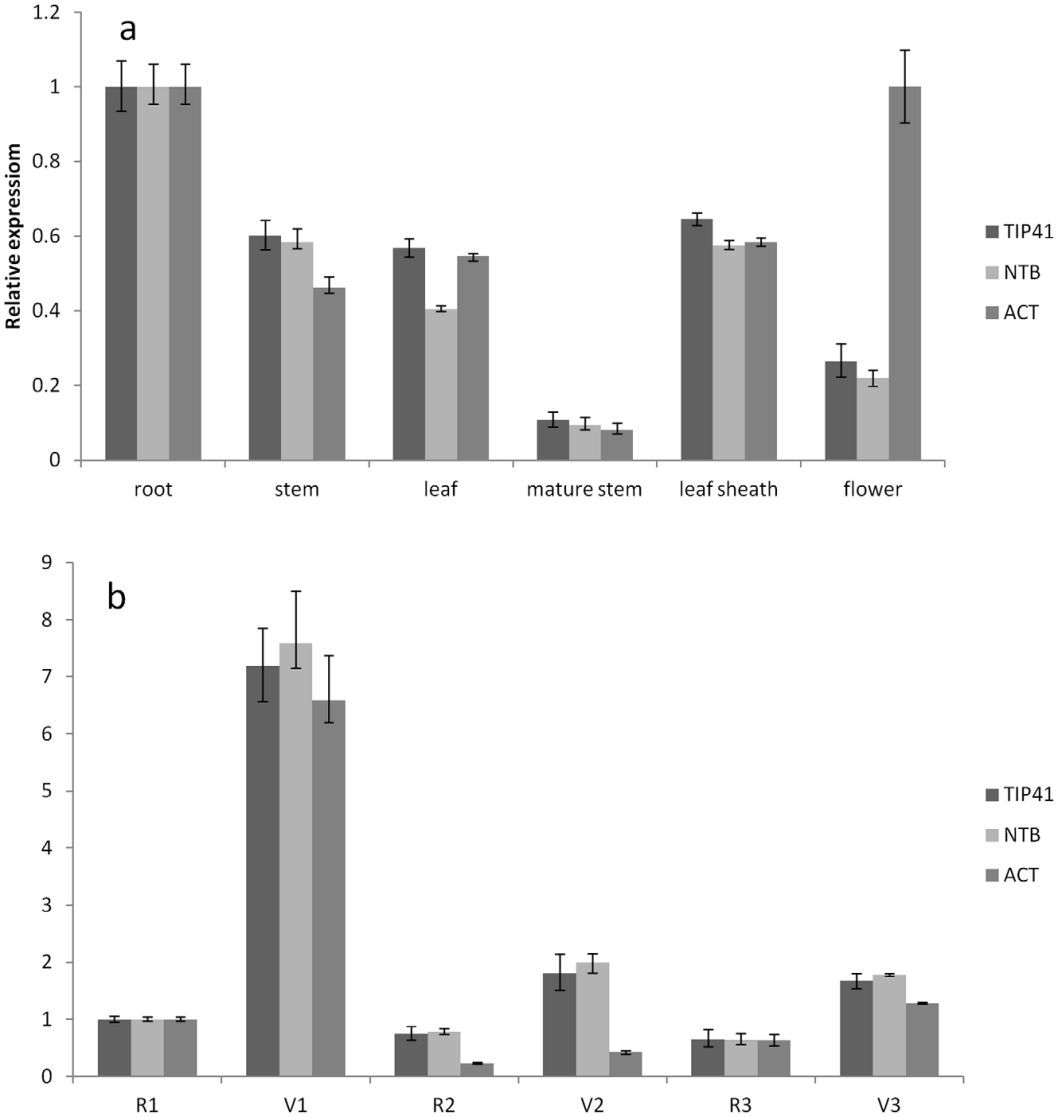
Expression levels of *AP2-like*. (a)Different tissues and (b)Various development stages exposed to different environments in three locations (1–3). V: vegetative stage; R: reproductive stage. Gene was normalized to individual and/or combined reference genes. Error bars show the standard error calculated from three biological replicates.

*APETALA2(AP2)-*like gene which mainly acts as flowering reprecessor [18] was selected to further evaluate the reliability of the reference genes by (RT-)qPCR. Relative expression of *AP2-like* was calculated by using *TIP41* or *NTB* as internal controls. The highest expression level of the *AP2-*like gene was found in root and the lowest level was in mature stem and flower (Figure3a). The expression profiles of the *AP2-*like gene obtained using these two reference genes showed a similar trend in all different tissues, developmental stages and sampling locations (Figure 3b), further demonstrating that both *TIP41* and *NTB* were adequate internal controls. When *ACT*, an unstably expressed gene (Table 2), was used as the reference for normalization, the expression profile of *AP2*-like gene changed, i.e., *AP2*-like expression were over estimated in flower (Figure 3a) and under estimated in samples collected from Location-2 (Figure 3b). Figure 3b also showed that expressions of some bamboo genes (e.g., *AP2*-like and *ACT*) were affected by environmental factors represented as sampling locations.

## Discussion

The invention of (RT-)qPCR has significantly improved the detection and quantification of expression profiles of selected genes in distinct biological samples. The main advantages of this technique are its high sensitivity, high specificity and broad quantification range [19], [20]. Due to its accuracy, simplicity and sensitivity for quantifying gene transcript levels across a broad range, (RT-)qPCR is the first choice for detection and quantification of gene expression profiles. For valid (RT-)qPCR analysis, an appropriate internal control gene is required. The ideal control gene should have relatively stable expression in distinct biological samples, which can be from different cell types, developmental stages, and from samples exposed to different experimental conditions.

Bamboo represents the only major lineage of grasses, which is also one of the fastest-growing plant species and exhibits monocarpic mass flowering and death after a very long vegetative phase [21]. These characteristics are quite different from those of other woody plants (e.g. *Populus*) and grasses (e.g. rice). So far, only a few studies have focused on the molecular mechanisms controlling flowering and rapid growth in bamboo. Once the entire bamboo genome sequencing is completed, most of the genes related to flowering and rapid growth can be identified and further functional annotation and analysis can be performed. Therefore, it is necessary to select a reference gene that is stably expressed.

To evaluate the best sets of reference genes for different organs and developmental stages in bamboo, three different statistical approaches, geNorm, NormFinder and BestKeeper, were utilized to identify the stability of expression of 14 candidate genes. Inconsistency between the three methods is expected, since they are based on distinct statistical algorithms. However, *TIP41, CYP, NTB* and *CAC* were identified as the most stable genes in all samples by the three methods used in this study. *TIP41* was identified as one of the top-ranked reference genes in previous studies focusing on the selection of reference genes in both vegetative and reproductive stages of *Brassica juncea* and *Brassica Napus* [12]. Similarly, *TIP41* was also identified as the most stable reference gene for a vegetative sample of tomato [22]. The other stably expressed genes *CYP*, *NTB* and *CAC* were identified as the most stable reference genes in some other species [10], [22].

It is surprising that most of the popular reference genes performed poorly as reference genes in this study. Previously, *ACT* has been considered to be one of the best reference genes for assessing gene expression in many plant tissues and under different experimental conditions. Therefore, *ACT* has been used as an internal control in gene expression profiling studies in different organs and under experimental conditions in bamboo [23], [24], [25]. In the present analysis, *ACT* was not the best reference gene in bamboo, both in different tissues and at different developmental stages. The main reasons for this discrepancy may be that the *ACT* product not only acts as a form of filament providing cells with mechanical support and driving forces for movement, but also contributes to biological processes such as sensing environmental stimuli, internalizing membrane vesicles and moving over surfaces [26]. This result confirmed the necessity to evaluate reference genes in each experimental setting. *TUB* has also been widely used as a reference gene in gene expression studies in water lily and soybean [27], [28]. However, some studies revealed that *TUB* did not satisfy certain basic requirements for use as an internal control [29]. In this study, the results showed that *TUBα* and *TUBβ* were the less stably expressed genes, and therefore they were not the most reliable internal controls for comparative gene expression analysis. This may partly be explained by the fact that *TUB* not only acts as one of the major components of cytoskeletal structure, but also participates in other cellular functions [30]. Apart from the genes discussed above, *GAPDH, 18S* and *UBQ,* which act as housekeeping genes and are widely used in many research fields as reference genes, were also not suitable reference genes in bamboo.

A consensus was produced that *TIP41* and *NTB* were the most reliably expressed genes tested in bamboo, although slight difference on ranking order was shown by using the three popular programs (geNorm, NormFinder and Bestkeeper). Such a difference is mainly due to the different statistical algorithms used in these programs [31]. It has also been observed in selecting reference genes in tung tree during seed development [32] and in *citrus* trees subjected to different experimental conditions [33].

To validate the feasibility of using the reference genes selected in this study, the expression profile of *APETALA2(AP2)* was assessed in different tissues and in leaf samples collected from different sites at vegetative and reproductive stages. It is known that,in flowering initiation and development, *AP2* transcript factor is to repress flowering and flower development by inhibiting the expression of key regulatory genes like *SOC1*, *AGAMOUS*, *AGAMOUS-LIKE15 (AGL15)* through involvement of miRNAs [18], [34], [35], [36]. Recent studies in maize, rice, and barley have shown that miR172 and its target, the *AP2-*like genes are important in regulating phase transition and in the determination of floral organ identity in monocotyledons [37], [38], [39]. This study showed that the bamboo *AP2-*like gene exhibited a similar pattern with low expression levels at reproductive stage and high expression levels in vegetative stage (Figure 3b) using *TIP41* and *NTB* as internal controls. In addition, the bamboo *AP2-*like gene was expressed differentially in different tissues (Figure 3a) and could be affected by environmental factors (Figure 3b). Furthermore, the use of the stable reference genes or their combination resulted in the consistency of the relative transcript abundance of *AP2-*like gene, in contrast to that when the less stable reference gene *ACT* was used as internal control. The results clearly showed that using an unstable reference gene (e.g., *ACT*, Figure 3) generated biases that could lead to misinterpretation of gene expression patterns.

The genes evaluated in this study will be very useful for further gene expression analysis in different organs in bamboo, especially in gene expression analysis related to bamboo flowering and for characterization of gene functions. Moreover, this study provides useful guidelines for reference gene selection for researchers working on other bamboo species.

## Materials and Methods

### Plant Materials and Biological Samples

The *P. edulis* samples were different tissues collected from plants at different development stages, grown in three different districts, Yezhudian Forest Farm (N25°13′24.38″,E110°43′13.84″, location-1) in Guanyang County, Haiyangshan Forest Farm (N25° 12′23.33″,E110°43′13.84″, location-2) in Lingchuan County and Dupengling Forest Farm (N25°01′11.43″,E110°43′14.09″, location-3) in Gongcheng County in Guangxi Zhuang Autonomous Region, China. The farms are local government-owned, no specific permits were required for research and the field studies did not involve any protected species. After collection, samples were immediately frozen in liquid nitrogen and stored at −80°C until further use. For samples from different tissues, root, stem, leaves without the leaf sheath and leaf sheath samples were collected from 3-month-old bamboo seedlings grown in a greenhouse at 20–25°C in the Chinese Academy of Forestry. The mature stems were gathered from two-year-old bamboo shoots and the flowers were harvested in Guilin. For samples at different developmental stages, leaves were collected from bamboo at vegetative and reproductive stages, grown in three districts in Guilin. All samples were collected and processed in sets of three replicates.

### RNA Extraction and cDNA Synthesis

Total RNA was extracted from the collected tissues and leaves from different developmental stages according to the manufacturer’s instructions (mirVana RNA Isolation Kit, Ambion). The integrity of RNA samples was evaluated by using an Agilent 2100 Bioanalyzer (Agilent Technologies) and the purity/concentration was determined using a NanoDrop 8000 spectrophotometer (NanoDrop, Thermo Scientific). Samples with concentrations greater than 100 ng.ml^−1^ and an optical density absorption ratio A260/A280 greater than 1.8 were used for cDNA synthesis. Individual RNA samples were stored at −70°C and then 2 µg of total RNA were used as template in RT reactions with the SuperScript III reverse transcriptase (Invitrogen), according to the manufacturer’s instructions. Before each qPCR stage, cDNA products were diluted 20-fold prior to use in real-time PCR. To ensure the absence of contamination or primer-dimer formation for each primer pair, a template-free control reaction was run. Absence of genomic DNA contamination was tested by PCR using primers (5′-CTCTTCGGCCAGAAGCCAGTCAC-3′ and 5′-TTGGCACCACCCTTCAAGTGAGC-3′) designed to amplify an intron sequence of a reference gene, *GAPDH* (gi|242389794).

### Reverse Transcription Quantitative Real-time PCR

The primers for the 14 reference genes from *P. edulis* were designed by using the Primer 3 software (http://www.genome.wi.mit.edu/cgi-bin/primer/primer3.cgi). All primer pairs were initially tested via standard RT-PCR using the Premix Ex Taq (TaKaRa, Japan) and the presence of a single amplification product of the expected size for each gene was verified by electrophoresis on a 2.5% agarose gel, followed by staining with ethidium bromide. (RT-)qPCR reactions were carried out in 96-well blocks with an Applied Biosystems 7500 Real-Time PCR system using SYBR® Premix Ex Taq™ kit (TaKaRa, Japan). The reaction conditions were those recommended by the manufacturer (30 s at 95°C, 40 cycles of 95°C for 5 s, and 60°C for 34 s). The dissociation curve was obtained by heating the amplicon from 60 to 95°C. All (RT-)qPCR reactions were carried out in triplicate, both technical and biological. The final quantification cycle (Cq) values were the mean of nine values (biological triplicate, each in technical triplicate).

### Statistical Analyses

To select a suitable reference gene, the stability of mRNA expression of each candidate gene was statistically analyzed with three different types of Microsoft Excel-based software packages: geNorm, NormFinder, and BestKeeper. All three software packages were used according to the manufacturer’s instructions. Quantities of standard RNA were prepared by diluting cDNA (1, 1/5, 1/25, 1/125, 1/625, 1/3125; each gene sample in triplicate). Only Cq values of less than 40 were used to calculate correlation coefficients (R^2^ values) and amplification efficiencies (E) from the slope generated in Microsoft Excel 2003 according to the equation E =  [5^(1/slope)^–1] × 100%. All PCR assays showed efficiency values between 95% and 105%.
